# The *junctional protein associated with coronary artery disease* predicts adverse cardiovascular events in patients with acute coronary syndromes at high residual risk

**DOI:** 10.1093/eurheartj/ehaf979

**Published:** 2025-12-23

**Authors:** Simon Kraler, Luca Liberale, Amedeo Tirandi, Margherita Moriero, Yifan Wang, Mohamed Farag, Federico Carbone, Maria B Bertolotto, Valentina Pusterla, Davide Ramoni, Stefano Ministrini, Yustina M Puspitasari, Francesco Bruno, Lorenz Räber, Davide Di Vece, Christian Templin, Olivier Muller, François Mach, Filippo Crea, Giovanni G Camici, Tetiana Lapikova-Bryhinska, Alexander Akhmedov, Arnold von Eckardstein, Diana A Gorog, Fabrizio Montecucco, Thomas F Lüscher

**Affiliations:** Center for Molecular Cardiology, University of Zurich, 8952 Schlieren, Switzerland; Department of Cardiology, Swiss Heart Center, Inselspital Bern, 3010 Bern, Switzerland; First Clinic of Internal Medicine, Department of Internal Medicine, University of Genoa, Genoa, Italy; IRCCS Ospedale Policlinico San Martino Genoa—Italian Cardiovascular Network, Genoa, Italy; Center for Molecular Cardiology, University of Zurich, 8952 Schlieren, Switzerland; First Clinic of Internal Medicine, Department of Internal Medicine, University of Genoa, Genoa, Italy; First Clinic of Internal Medicine, Department of Internal Medicine, University of Genoa, Genoa, Italy; Center for Molecular Cardiology, University of Zurich, 8952 Schlieren, Switzerland; Department of Cardiology, East and North Hertfordshire NHS Trust, Coreys Mill Lane, Stevenage SG1 4AB, UK; School of Life and Medical Sciences, Postgraduate Medical School, University of Hertfordshire, Hertfordshire, UK; First Clinic of Internal Medicine, Department of Internal Medicine, University of Genoa, Genoa, Italy; IRCCS Ospedale Policlinico San Martino Genoa—Italian Cardiovascular Network, Genoa, Italy; First Clinic of Internal Medicine, Department of Internal Medicine, University of Genoa, Genoa, Italy; First Clinic of Internal Medicine, Department of Internal Medicine, University of Genoa, Genoa, Italy; First Clinic of Internal Medicine, Department of Internal Medicine, University of Genoa, Genoa, Italy; Center for Molecular Cardiology, University of Zurich, 8952 Schlieren, Switzerland; Department of Internal Medicine, Limmattal Hospital, Urdorferstrasse 100, 8952 Schlieren, Switzerland; Center for Molecular Cardiology, University of Zurich, 8952 Schlieren, Switzerland; Division of Cardiology, Cardiovascular and Thoracic Department, Città della Salute e della Scienza, 10126 Turin, Italy; Royal Brompton and Harefield Hospitals, Sydney St, London SW3 6NP, UK; Department of Cardiology, Swiss Heart Center, Inselspital Bern, 3010 Bern, Switzerland; First Clinic of Internal Medicine, Department of Internal Medicine, University of Genoa, Genoa, Italy; Department of Internal Medicine B, University Medicine Greifswald, 17475 Greifswald, Germany; Department of Internal Medicine B, University Medicine Greifswald, 17475 Greifswald, Germany; Department of Cardiology, Lausanne University Hospital-CHUV, 1005 Lausanne, Switzerland; Department of Cardiology, University Hospital Geneva, 1205 Geneva, Switzerland; Department of Cardiovascular and Pulmonary Sciences, Catholic University of the Sacred Heart, Rome, Italy; Center of Excellence of Cardiovascular Sciences, Ospedale Isola Tiberina—Gemelli Isola, Rome, Italy; Center for Molecular Cardiology, University of Zurich, 8952 Schlieren, Switzerland; Center for Molecular Cardiology, University of Zurich, 8952 Schlieren, Switzerland; Center for Molecular Cardiology, University of Zurich, 8952 Schlieren, Switzerland; Institute of Clinical Chemistry, University Hospital Zurich, 8091 Zurich, Switzerland; Department of Cardiology, East and North Hertfordshire NHS Trust, Coreys Mill Lane, Stevenage SG1 4AB, UK; School of Life and Medical Sciences, Postgraduate Medical School, University of Hertfordshire, Hertfordshire, UK; Royal Brompton and Harefield Hospitals, Sydney St, London SW3 6NP, UK; National Heart and Lung Institute, Imperial College, Dovehouse Street, London SW3 6LY, UK; School of Cardiovascular Medicine and Sciences, Kings College London, The James Black Centre, 125 Coldharbour Ln, London SE5 9NU, UK; First Clinic of Internal Medicine, Department of Internal Medicine, University of Genoa, Genoa, Italy; IRCCS Ospedale Policlinico San Martino Genoa—Italian Cardiovascular Network, Genoa, Italy; Center for Molecular Cardiology, University of Zurich, 8952 Schlieren, Switzerland; Royal Brompton and Harefield Hospitals, Sydney St, London SW3 6NP, UK; National Heart and Lung Institute, Imperial College, Dovehouse Street, London SW3 6LY, UK; School of Cardiovascular Medicine and Sciences, Kings College London, The James Black Centre, 125 Coldharbour Ln, London SE5 9NU, UK

**Keywords:** Acute coronary syndromes, Atherosclerosis, Residual risk, Inflammation, Lipids, hs-CRP, LDL-c, JCAD, KIAA1462, Junctional protein associated with coronary artery disease

## Abstract

**Background and Aims:**

Patients with acute coronary syndromes (ACS) are at high ischaemic risk to which cholesterol, inflammation, and yet-to-be-identified pathways jointly contribute. The *junctional protein associated with coronary artery disease* (JCAD) drives incident cardiovascular events by acting on coagulation and fibrinolysis. This study aimed to assess whether JCAD serves as a novel marker of or target to address residual risk.

**Methods:**

In the discovery cohort (SPUM-ACS; *n* = 4787), ACS patients at residual lipid risk [RLR; on-statin LDL cholesterol (LDL-c) ≥70 mg/dL or ≥1.8 mmol/L], residual inflammatory risk [RIR; on-statin high-sensitivity C-reactive protein (hs-CRP) ≥2.0 mg/L], or both (RILR; on-statin LDL-c ≥70 mg/dL and hs-CRP ≥2.0 mg/L) were identified and compared with propensity-score matched controls. Contributions of hs-CRP, LDL-c and JCAD to recurrent major adverse cardiovascular events (MACE) were analysed. In an independent cohort (RISK-PPCI study; *n* = 496), effects of JCAD on endogenous coagulation and fibrinolysis were gauged, and JCAD–MACE associations were externally validated.

**Results:**

At 1 year, patients at RLR, RIR, or RILR were at higher MACE risk as compared to controls [hazard ratio (HR), 1.55, 95% confidence interval (CI) 1.08–2.23; HR 1.80, 95% CI 1.24–2.61; and HR 1.75, 95% CI 1.12–2.75, respectively]. In those at RLR, MACE risk rose with increasing hs-CRP and JCAD, respectively, in uni- (HR per log_2_ increase, 1.17, 95% CI 1.06–1.30; HR 1.29, 95% CI 1.03–1.62) and multivariable-adjusted models [adjusted (a)HR 1.16, 95% CI 1.03–1.30; aHR 1.27, 95% CI 1.01–1.60]. In those at RIR, MACE risk increased 1.28-fold per log_2_ increase in JCAD (HR 1.28, 95% CI 1.03–1.59), which prevailed in multivariable-adjusted models (aHR 1.31, 95% CI 1.04–1.65). Similarly, in patients at RILR, MACE risk increased almost linearly with increasing JCAD (HR 1.45, 95% CI 1.09–1.92), independently of potential confounders (aHR 1.47, 95% CI 1.11–1.97). Plasma levels of JCAD correlated positively with proxies of impaired endogenous fibrinolysis, with the JCAD–MACE association being similarly observed in the external validation cohort.

**Conclusions:**

Acute coronary syndrome patients at RLR, RIR, or both are at high ischaemic risk. By modulating coagulation and endogenous fibrinolysis, JCAD represents a promising candidate to address the high residual risk that persists in ACS patients receiving guideline-recommended care.

**ClinicalTrials.gov Identifiers:**

NCT01000701, NCT02562690

## Structured Graphical Abstract

**Structured Graphical Abstract:**
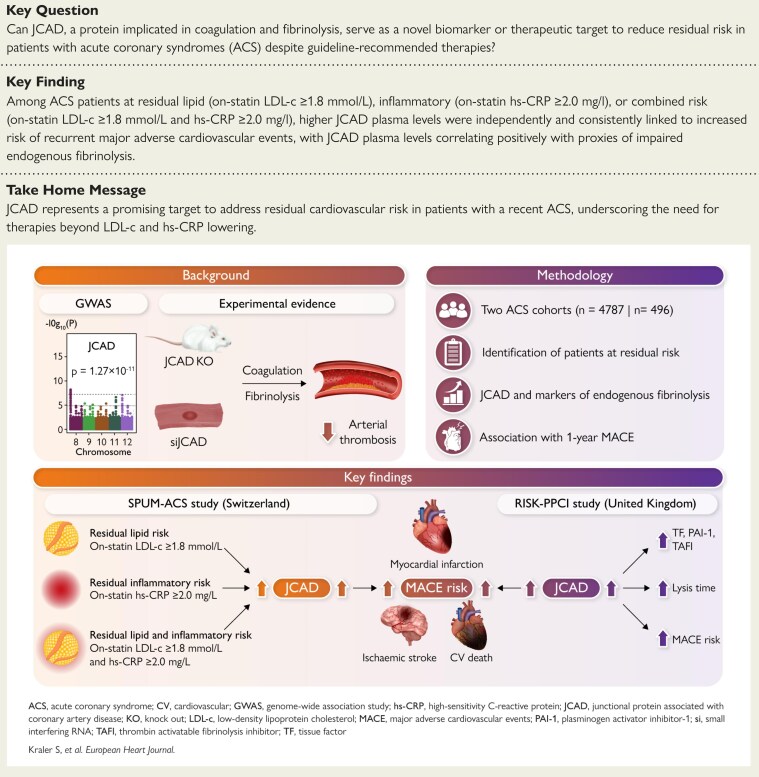
Genomic and experimental evidence implicate the *junctional protein associated with coronary artery disease* (JCAD) in cardiovascular disease. In patients with ACS from the SPUM-ACS cohort (*n* = 4787), those with residual lipid, inflammatory, or combined risk showed higher cumulative incidence of major adverse cardiovascular events (MACE). Elevated plasma levels of JCAD independently predicted MACE across all residual risk groups and correlated positvely with impaired fibrinolysis in an external validation cohort (RISK-PPCI; *n* = 496). JCAD is a novel marker of and potential therapeutic target for residual risk after a recent ACS. ACS, acute coronary syndromes; GWAS, genome-wide association study; hs-CRP, high-sensitivity C-reactive protein; LDL-c, LDL cholesterol; MACE, major adverse cardiovascular events; PAI-1, plasminogen activator inhibitor-1; si, small interfering RNA; TAFI, thrombin activatable fibrinolysis inhibitor; TF, tissue factor.

**See the editorial comment for this article ‘The return of procoagulation: junctional protein JCAD', by A.S. Jaffe, https://doi.org/10.1093/eurheartj/ehag024.**

## Introduction

Owing to the broad implementation of early revascularization strategies combined with highly effective secondary prevention measures, outcomes of patients with acute coronary syndromes (ACS) have improved steadily over the last decades.^1–8^ Nonetheless, a considerable proportion of ACS patients receiving guideline-recommended care remains at high residual cardiovascular risk, to which cholesterol, inflammation, and yet-to-be-identified pathways jointly contribute.^9–11^

As early as 1994, the landmark 4S trial provided strong evidence that statin-induced LDL cholesterol (LDL-c) lowering over a 6-year period results in a substantial risk reduction of major adverse cardiovascular events (MACE) when compared with placebo.^12^ More than two decades later, stimulated by the discovery of proprotein convertase subtilisin/kexin type 9 (PCSK9),^13,14^ the FOURIER trial showed that the ischaemic risk can be further reduced by roughly 15% through aggressive LDL-c lowering by PCSK9 inhibition on a background of statin therapy.^15^ Similar results were obtained in the more recently conducted ODYSSEY OUTCOMES trial.^16^

Almost simultaneously, based on experimental and clinical data,^17–19^ the CANTOS and subsequently the COLCOT and LoDoCo trials showed that anti-inflammatory remedies acting downstream of the NLRP3 inflammasome provided additional clinical benefit.^8^ In CANTOS, patients with a recent ACS randomized to 150 mg canakinumab (a monoclonal antibody targeting interleukin-1β) experienced a roughly 15% risk reduction in MACE when compared with placebo over a median follow-up of 3.7 years, notably independent of lipid-level lowering.^5^ Similarly, in stabilized ACS patients, colchicine 0.5 mg daily led to a 23% relative risk reduction of MACE in COLCOT^7^ and 31% in LoDoCo2.^6^ Yet, the recently published CLEAR-SYNERGY trial does not support the use of this non-specific anti-inflammatory agent in the acute setting.^20,21^ Although its interpretation is limited by several factors—including early drug administration, high treatment discontinuation, recruitment of mainly ST-elevation myocardial infarction (STEMI) patients, and challenges related to the COVID-19 pandemic—the lack of therapeutic benefit reinforces the need to explore alternative and more specific targets in this high-risk population.^21,22^

Initially identified by genome-wide association studies (GWAS),^23,24^ the *junctional protein associated with coronary artery disease* (JCAD) drives arterial thrombus formation and incident cardiovascular events independently of traditional risk factors.^23,24^ As suggested by experimental studies,^25^ this may occur via the modulation of coagulation and fibrinolysis, the latter being strongly linked to ischaemic events and residual risk in patients with a recent ACS.^10^

Herein, we aimed to characterize ACS patients at residual lipid risk (RLR), residual inflammatory risk (RIR), or both (RILR), and to define the role of JCAD as a potential mediator of the persisting ischaemic risk in patients with a recent ACS.

## Methods

### Study participants

The SPUM-ACS study is a multicentre, prospective cohort study in which a total of 4787 patients with a main diagnosis of ACS were recruited, as described previously.^26–33^ Briefly, patients aged ≥18 years with a main diagnosis of ACS presenting to one of the four major university hospitals in Switzerland (Zurich, Bern, Geneva, and Lausanne) were included. Patients with severe physical disability, dementia, or life expectancy <1 year (for non-cardiac reasons) were not eligible for inclusion. RLR was defined as on-treatment LDL-c ≥70 mg/dL (≥1.8 mmol/L), while RIR was defined as on-treatment high-sensitivity C-reactive protein (hs-CRP) ≥2.0 mg/L at the time of initial presentation, as reported previously.^34^ Patients meeting both criteria [on-treatment LDL-c ≥70 mg/dL (≥1.8 mmol/L) and hs-CRP ≥2.0 mg/L] were classified as being at residual RILR. The RISK-PPCI study is a single-centre (Lister Hospital, Hertfordshire, UK), prospective cohort study in which ACS patients undergoing thrombotic status assessment prior to primary percutaneous coronary intervention (PCI) were recruited, with its study design and in- and exclusion criteria being detailed elsewhere.^10^ In brief, consecutive patients presenting with STEMI were eligible for study inclusion. RISK-PPCI study participants were excluded if they were already on oral anticoagulation, had known coagulation disorders, sepsis, platelet count <10^8^/µL, haemoglobin <8 g/dL, active malignancy, or were unable to take dual antiplatelet therapy. Patients included in SPUM-ACS and RISK-PPCI were treated according to current guideline recommendations, which includes a loading dose of antithrombotic therapy prior to coronary angiography. All study participants provided written informed consent; a deferred consent strategy was used in RISK-PPCI study participants. Study protocols adhered to the Declaration of Helsinki and were approved by the institutional review boards.

### Quantification of biomarkers and proxies of coagulation and fibrinolysis

Levels of JCAD and hs-CRP levels were assessed in EDTA (ethylenediaminetetraacetic acid)-plasma samples obtained prior to any coronary intervention. For the quantification of JCAD, commercially available enzyme-linked immunosorbent assays following the manufacturers’ instructions were used (MyBiosource, San Diego, CA, USA), with intra- and interassay coefficients of variation being <15%, as reported.^25,35^ For the assessment of hs-CRP, a particle-enhanced turbidimetric immunoassay was employed (Roche Diagnostics, Boehringer Mannheim, Indianapolis, IN, USA), as reported.^26^ Similarly, tissue factor (TF), plasminogen activator inhibitor (PAI)-1 (both obtained from R&D Systems, Minneapolis, MN, USA), and thrombin activatable fibrinolysis inhibitor (TAFI) quantification was done by means of enzyme-linked immunosorbent assays (MyBiosource, San Diego, CA, USA), with intra- and interassay coefficients of variation being <15%, as reported.^25^ Standard lipid panels were measured in all patients,^28^ and LDL-c levels were calculated using the Sampson equation.^28,36^ In RISK-PPCI study participants, native non-anticoagulated blood drawn prior to PCI was subjected to a validated, point-of-care global thrombosis test (GTT) (Thromboquest Ltd, London, UK), as described.^37^ Briefly, the blood sample was introduced into the GTT cartridge and endogenous lysis time, i.e. the time required for flow restoration after an occlusive thrombus as formed under high-shear stress, was measured. Inter- and intra-assay coefficients of variation were determined by analysing native blood samples from 10 stable patients on 2 occasions, 48 h apart, with all samples being processed simultaneously. Study personnel involved in the biomarker measurements were fully blinded to study participants’ baseline and outcome data.

### Clinical follow-up, adjudication of adverse events, and study oversight

SPUM-ACS study participants were followed prospectively up to 1 year (clinical visit). Trained study personnel documented baseline data at each study site using a centralized data entry system (CARDIOBASE, Clinical Trial Unit and Department of Cardiology, University Hospital Bern, Bern, Switzerland and Webspirit Systems GmbH, Ulm, Germany). All adverse events of the primary and secondary endpoints of the present study were adjudicated by an independent clinical endpoint committee consisting of three expert cardiologists blinded to study participants’ baseline characteristics using pre-specified adjudication forms. Among RISK-PPCI study participants, study-specific case record forms were completed during the index admission, with patients being followed over a 1-year period, as previously reported.^10^ Patient recruitment, biomarker measurements, and the collection of baseline and event data were overseen by a study committee involving expert cardiologists from each participating study centre.

### Definition of the primary endpoint and main study objectives

The primary endpoint of the current study was MACE during 1-year of follow-up, defined as a composite measure of non-fatal myocardial infarction, non-fatal stroke, and cardiovascular death, whichever occurred first. The present study aimed to characterize the ischaemic risk of ACS patients at RLR, RIR, and RILR, and to study independent associations between individual biomarker levels (i.e. LDL-c, hs-CRP and JCAD) and the primary endpoint. Secondary objectives included the study of JCAD plasma levels and their associations with proxies of endogenous coagulation/fibrinolysis.

### Statistical analysis

Continuous data are shown as median and interquartile range (IQR), and categorical data as counts and percentages (%). Patients were classified as being at RLR, RIR, or RILR (see Supplementary data online, *Figures S1* and *S2*).^34^ Control patients were identified by nearest neighbour (‘greedy’) matching in a 1:1 fashion. To mitigate a potential missing data bias (see Supplementary data online, *Table S1*), propensity score (PS) modelling was done on multiply imputed data (*n* = 20 data sets) within each dataset.^38,39^ The PS was derived from *a priori*-defined covariates linked to both group assignment and the primary endpoint, including sex, history of hypercholesterolaemia, GRACE risk scores, smoking history, a diagnosis of diabetes, a history of congestive heart failure, and presence of anterior myocardial infarction.^29,30,34,40^ To assess covariate balance, the standardized mean difference was used (*Table 1*).^28,41^ To yield most accurate standard errors following PS matching, time-to-event data were modelled using complex survey design-based Cox proportional hazard regression models, with estimates being pooled according to Rubin's rules. To plot the probability of MACE during follow-up, Nelson-Aalen curves for one randomly chosen dataset were plotted. To test the predictive utility of LDL-c, hs-CRP, and JCAD in patients at RLR, RIR, or RILR, uni- and multivariable-adjusted Cox proportional hazard regression models were fitted within each group accounting for potential confounders, as specified in the figure legends. In linear models, biomarker data were log_2_-transformed (i.e. one unit increase corresponds to a doubling in biomarker levels). To model nonlinear relationships of biomarker data with the primary endpoint, restricted cubic splines were used, with knots fixed at the 25th, 50th, and 75th percentiles. Discrimination was quantified with Harrell’s concordance index. Model adequacy penalizing complexity was assessed with the Akaike information criterion (AIC), reporting ΔAIC vs baseline and performing likelihood-ratio *χ*^2^ tests for nested comparisons. We adhered to the principles outlined by the STROBE initiative and followed the AHA Scientific Publication Committee’s recommendations for statistical reporting.^42,43^ A two-tailed *P* < .05 was deemed statistically significant throughout. All analyses were conducted in R version 4.2.3 (R Foundation for Statistical Computing, Vienna, Austria).

**Table 1:** Baseline characteristics of patients at residual lipid risk, inflammatory risk, and combined risk and propensity score-matched controls

	Not at RLR (*n* = 892)	RLR (*n* = 892)	SMD	Not at RIR (*n* = 683)	RIR (*n* = 683)	SMD	Not at RILR (*n* = 460)	RILR (*n* = 460)	SMD
Age ≥65 years	418 (46.9)	482 (54.0)	0.14	344 (50.4)	407 (59.6)	0.19	225 (48.9)	248 (53.9)	0.10
Female	193 (21.6)	186 (20.9)	0.02	155 (22.7)	152 (22.3)	0.01	114 (24.8)	113 (24.6)	0.01
GRACE risk (%)^a^	1.88 [0.98–3.79]	1.86 [0.96–3.71]	0.02	2.04 [1.06–4.13]	2.08 [1.07–4.29]	0.02	1.96 [0.97–3.88]	1.97 [0.98–3.87]	0.03
Anterior MI	80 (9.0)	86 (9.6)	0.02	51 (7.5)	54 (7.9)	0.02	37 (8.0)	36 (7.8)	0.01
LVEF	50.00 [45.00–60.00]	55.00 [45.00–60.00]	0.06	50.00 [45.00–60.00]	55.00 [45.00–60.00]	<0.01	52.50 [45.00–60.00]	55.00 [45.00–60.00]	0.02
SBP (mmHg)	129.00 [114.00–144.00]	130.00 [114.00–145.00]	0.04	128.00 [113.00–142.00]	130.00 [115.00–144.00]	0.09	129.00 [113.00–143.00]	130.00 [115.00–145.00]	0.09
BMI (kg/m^2^)	26.90 [24.40–29.95]	27.00 [24.70–29.70]	0.03	26.80 [24.20–30.00]	27.40 [24.70–30.90]	0.11	26.70 [24.20–30.00]	27.20 [24.70–30.40]	0.06
BSA^b^ (m^2^)	1.93 [1.80–2.06]	1.94 [1.79–2.06]	0.01	1.94 [1.81–2.05]	1.93 [1.79–2.06]	0.03	1.94 [1.79–2.06]	1.92 [1.77–2.06]	0.04
Hx of smoking	616 (69.9)	614 (70.1)	<0.01	471 (69.9)	463 (69.5)	0.01	330 (72.2)	328 (72.9)	0.02
≥1 drink per day	268 (32.8)	281 (35.8)	0.07	228 (36.6)	213 (36.3)	0.01	151 (35.6)	145 (36.5)	0.02
eGFR^c^ (mL/min/1.73 m^2^)	87.43 [70.98–98.69]	84.52 [68.86–96.03]	0.10	87.33 [71.53–96.43]	81.55 [61.62–94.38]	0.27	88.50 [71.85–97.68]	83.81 [66.25–96.40]	0.17
Hx of DM	215 (24.1)	222 (24.9)	0.02	239 (35.0)	240 (35.1)	0.00	124 (27.0)	124 (27.0)	<0.01
FHx of CAD	209 (23.6)	247 (28.0)	0.10	163 (24.2)	171 (25.2)	0.02	105 (22.9)	120 (26.2)	0.08
Hx of PAD	57 (6.4)	92 (10.3)	0.14	51 (7.5)	98 (14.3)	0.22	32 (7.0)	58 (12.6)	0.19
Hx of stroke/TIA	33 (3.7)	61 (6.8)	0.14	34 (5.0)	59 (8.6)	0.15	17 (3.7)	38 (8.3)	0.19
Hx of HF	21 (2.4)	21 (2.4)	<0.01	17 (2.5)	18 (2.6)	0.01	11 (2.4)	12 (2.6)	0.01
Hx of dialysis	5 (0.6)	8 (0.9)	0.04	2 (0.3)	10 (1.5)	0.13	1 (0.2)	5 (1.1)	0.11
Hx of malignancy	83 (9.3)	80 (9.0)	0.01	54 (7.9)	71 (10.4)	0.09	44 (9.6)	37 (8.1)	0.05
(D)OAC	36 (6.3)	66 (7.4)	0.04	28 (6.0)	69 (10.1)	0.15	22 (7.1)	44 (9.6)	0.09
SAPT + (D)OAC	13 (2.3)	31 (3.5)	0.07	8 (1.7)	36 (5.3)	0.19	7 (2.2)	21 (4.6)	0.13
Statin (highest dose)^d^	59 (6.6)	322 (36.1)	0.77	59 (8.6)	248 (36.3)	0.70	53 (11.5)	169 (36.7)	0.62
Statin (any) + other lipid-lowering drugs (any)	14 (2.5)	43 (4.8)	0.13	8 (1.7)	44 (6.4)	0.24	10 (3.2)	23 (5.0)	0.09
Statin (any) + ezetimib	14 (2.5)	41 (4.6)	0.12	8 (1.7)	42 (6.1)	0.23	10 (3.2)	22 (4.8)	0.08
Statin (highest dose) + other lipid-lowering drugs (any)	9 (1.0)	22 (2.5)	0.11	4 (0.6)	23 (3.4)	0.20	5 (1.1)	13 (2.8)	0.13
Statin (highest dose) + ezetimib	9 (1.0)	21 (2.4)	0.11	4 (0.6)	21 (3.1)	0.19	5 (1.1)	12 (2.6)	0.11
NT-proBNP (ng/L)	380.00 [121.00–1267.00]	364.00 [133.00–1250.00]	0.01	308.00 [114.25–1124.50]	801.00 [267.25–2470.75]	0.31	339.00 [126.00–1366.00]	651.00 [243.25–2047.25]	0.18
hs-cTnT (ng/L)	204.00 [63.25–690.25]	132.00 [41.00–489.50]	0.11	159.00 [52.50–523.00]	216.00 [55.00–723.00]	0.12	156.00 [53.00–587.00]	217.00 [51.75–696.00]	0.06
Hb (g/dL)	14.00 [12.80–15.10]	13.70 [12.67–14.90]	0.07	14.00 [12.80–15.10]	13.30 [12.00–14.60]	0.03	14.00 [12.70–15.00]	13.50 [12.40–14.70]	0.06
HDL-c (mg/dL)	44.47 [36.74–54.14]	43.31 [36.35–52.59]	0.07	43.70 [36.74–54.14]	42.15 [34.42–51.43]	0.15	43.31 [35.96–53.75]	42.15 [35.19–51.43]	0.10
Triglycerides (mg/dL)	100.09 [66.43–148.80]	97.43 [65.54–147.92]	0.08	96.55 [67.32–147.03]	97.43 [65.54–145.26]	0.03	95.66 [66.65–141.50]	105.40 [70.64–152.57]	0.14
LDL-c ≥70 mg/dL	688 (80.9)	892 (100.0)	0.69	596 (90.4)	460 (70.1)	0.53	371 (83.7)	460 (100.0)	0.62
hs-CRP ≥2 mg/L	535 (65.2)	460 (56.0)	0.19	296 (48.8)	683 (100.0)	1.45	233 (56.4)	460 (100.0)	1.24
JCAD (ng/mL)	1.27 [0.55–1.94]	1.18 [0.46–1.88]	0.09	1.24 [0.60–1.93]	1.05 [0.41–1.91]	0.05	1.23 [0.50–1.88]	1.05 [0.42–1.92]	0.04

Continuous data are shown as median (25th–75th percentiles) and categorical data as counts (%). To convert cholesterol levels to millimoles per litre, multiply by 0.0259.

BMI, body mass index; BSA, body surface area; CAD, coronary artery disease; DM, diabetes mellitus; (D)OAC, (direct) oral anticoagulants; eGFR, estimated glomerular filtration rate; FHx, family history; GRACE, Global Registry of Acute Coronary Events; Hb, haemoglobin; HDL-c, HDL cholesterol; HF, heart failure; hs-CRP, high-sensitivity C-reactive protein; hs-cTnT, high-sensitivity cardiac troponin-T; Hx, history; JCAD, junctional protein associated with coronary artery disease; LDL-c, LDL cholesterol; LVEF, left-ventricular ejection fraction; MI, myocardial infarction; NT-proBNP, N-terminal pro–B-type natriuretic peptide; PAD, peripheral artery disease; PS, propensity score; RIR, residual inflammatory risk; RILR, residual inflammatory and lipid risk; RLR, residual lipid risk; SAPT, single antiplatelet therapy; SBP, systolic blood pressure; SMD, standardized mean difference; TIA, transient ischaemic attack.

^a^Refers to the in-hospital death endpoint.

^b^According to Du Bois and Du Bois.

^c^According to the CKD-EPI formula 2009.^44^

^d^Only available if type of statin was atorvastatin, rosuvastatin, fluvastatin, pravastatin, rosuvastatin, or simvastatin.

## Results

### Baseline characteristics of patients at residual risk

Among 4787 ACS patients recruited into SPUM-ACS, 892 were identified as being at RLR (on-statin LDL-c ≥70 mg/dL or ≥1.8 mmol/L), 683 at RIR (hs-CRP ≥2.0 mg/L), and 460 at RILR [LDL-c ≥70 mg/dL (≥1.8 mmol/L) and hs-CRP ≥2.0 mg/L] (see Supplementary data online, *Figures S1* and *S2*). Baseline characteristics of those at RLR, at RIR, and at RILR and their PS-matched controls are provided in *Table 1*. Among patients at RLR, 20.9% were female, with 54.0% being ≥65 years of age. About 10.3% of these patients had a history of peripheral artery disease (PAD), and 6.8% of stroke or transient ischaemic attack. Median hs-CRP levels were 2.5 (interquartile range, 1.00–7.50) mg/L, while plasma JCAD levels equalled 1.18 (0.46–1.88) ng/mL. Of all patients at RIR, 59.6% were ≥65 years of age, with 22.3% being female. Median estimated glomerular filtration rate (eGFR) was 81.6 (61.62–94.38) mL/min/1.73 m^2^,^44^ with 14.3% having a medical history of PAD. Median hs-CRP levels were 7.00 (3.55–19.20) mg/L, while JCAD plasma levels were 1.05 (0.41–1.91) ng/mL. Of all patients at RILR, 53.9% were ≥65 years of age, with 24.6% being female. Median eGFR was 83.81 (66.25–96.40) mL/min/1.73 m^2^, and 12.6% had a history of PAD. Median hs-CRP levels were 6.05 (3.50–16.22) mg/L, while median JCAD levels were 1.05 (0.42–1.92) ng/mL.

### Residual risk and major adverse cardiovascular events

**Figure 1:**
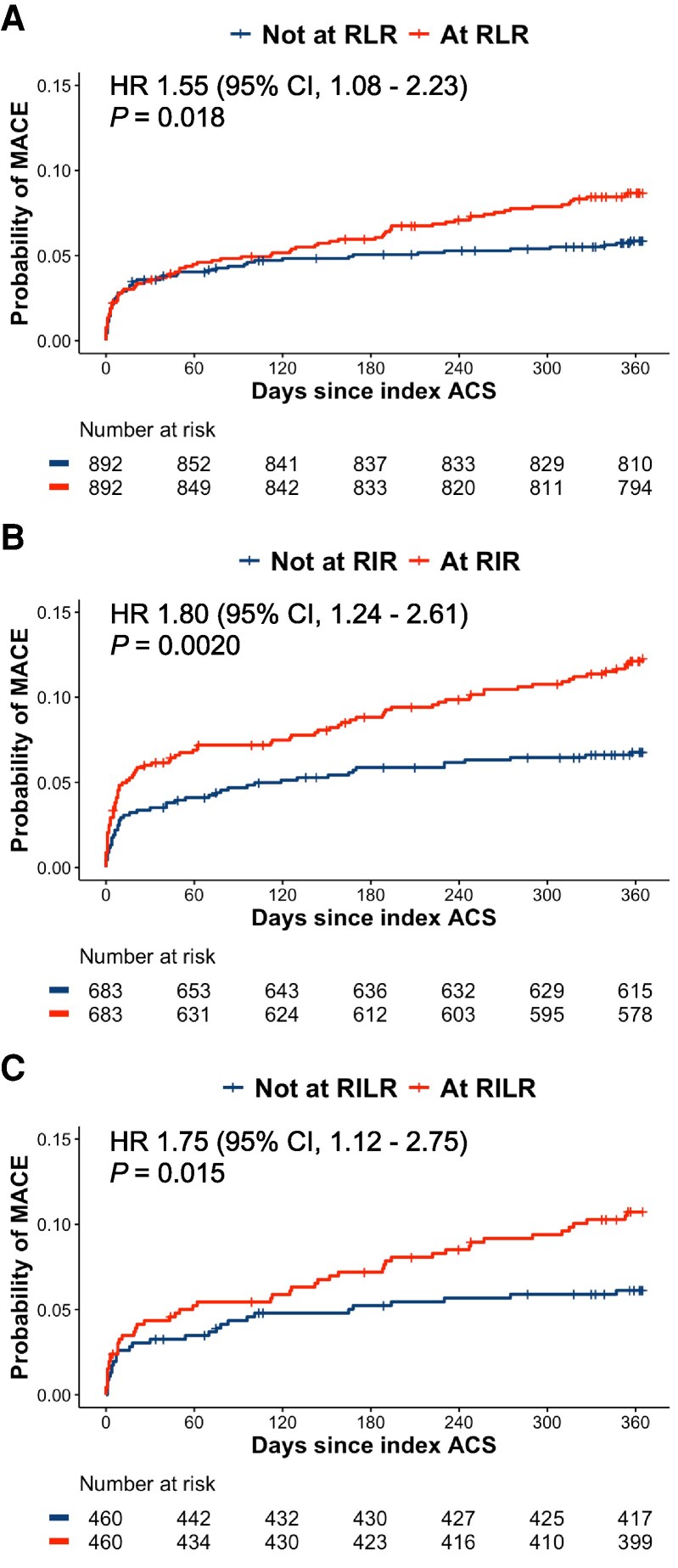
Risk of major adverse cardiovascular events among patients at residual risk relative to PS-matched controls. (*A*) RLR refers to residual lipid risk (on-statin LDL-c ≥70 mg/dL or ≥1.8 mmol/L). (*B*) RIR refers to residual inflammatory risk (on-statin hs-CRP ≥2.0 mg/L). (*C*) RILR refers to residual inflammatory and lipid risk (LDL-c ≥70 mg/dL or ≥1.8 mmol/L) and hs-CRP ≥2.0 mg/L). Right-censored observations are indicated as tick marks. The PS was calculated based on predefined covariates associated with both group assignment and ischaemic outcomes, including sex, history of hypercholesterolemia, GRACE risk scores, smoking status, presence of diabetes, history of congestive heart failure, and anterior myocardial infarction. Hazard ratios were obtained by complex-survey based proportional hazard regression models run on multiply imputed data (*n* = 20), with estimates being pooled according to Rubin's rules. ACS, acute coronary syndrome; CI, confidence interval; HR, hazard ratio; RIR, residual inflammatory risk; RLR, residual lipid risk; RILR, residual inflammatory and lipid risk; MACE, major adverse cardiovascular events

Among patients at RLR (on-statin LDL-c ≥70 mg/dL or ≥1.8 mmol/L), a total of 77 MACE occurred at 1 year, with a cumulative incidence of 8.67% [95% confidence interval (CI) 6.80–10.51]. When compared with PS-matched controls, patients at RLR had a 1.55-fold increased 1-year MACE risk [hazard ratio (HR) 1.55, 95% CI 1.08–2.23, *P* = .018] with survival curves starting to disperse as of 4 months after the index ACS (*Figure 1A*). In those at RIR (on-statin hs-CRP ≥ 2.0 mg/L), a total of 83 MACE occurred (cumulative incidence of 12.27%, 95% CI 9.76–14.71), transitioning into a HR of 1.80 (95% CI 1.24–2.61; *P* = .0020) for 1-year MACE (*Figure 1B*). Finally, in those at RILR [on-statin LDL-c ≥70.0 mg/dL (≥1.8 mmol/L) and hs-CRP ≥ 2.0 mg/L], 49 patients experienced MACE at 1 year, corresponding to a cumulative incidence of 10.72% (95% CI 7.84–13.52). Relative to PS-matched controls, these patients were at 1.75-fold increased MACE risk (HR 1.75, 95% CI 1.12–2.75; *P* = .015) (*Figure 1C*). Similar results were obtained when all patients not assigned to residual risk groups were used as controls (see Supplementary data online, *Figure S3* and *Table S2*).

### *Junctional protein associated with coronary artery disease* predicts major adverse cardiovascular events in patients at residual risk

**Figure 2:**
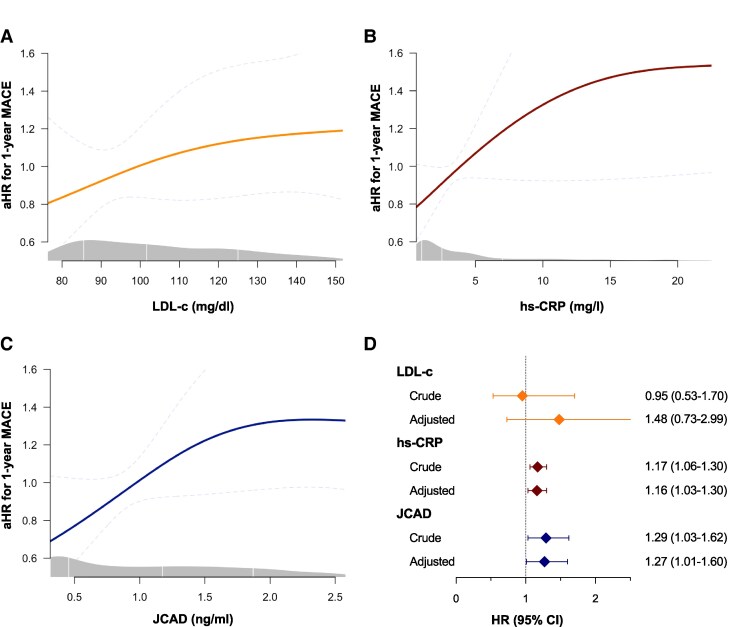
Risk of 1-year MACE according to biomarker levels of lipids, inflammation, and JCAD in patients at RLR (on-statin LDL-c ≥70 mg/dL or ≥1.8 mmol/L). Multivariable-adjusted three-knot restricted cubic spline curves (with knots fixed at the 25th, 50th, and 75th percentiles) on the associations between LDL-c (*A*; yellow), hs-CRP (*B*; red), and JCAD (*C*; blue) and 1-year MACE risk is shown. Crude and adjusted ratios of the hazard rates for each biomarker (mutually adjusted for each other) are shown in (*D*). Multivariable models include sex, age, JCAD, hs-CRP, and LDL-c. Biomarker data were log_2_-transformed. Note that tilted squares represent HR with line lengths corresponding to 95% confidence intervals. To convert cholesterol levels to millimoles per litre, multiply by 0.0259. aHR, adjusted hazard ratio; HR, hazard ratio; hs-CRP, high-sensitivity C-reactive protein; JCAD, junctional protein associated with coronary artery disease; LDL-c, LDL cholesterol; MACE, major adverse cardiovascular events

In those at RLR, LDL-c levels were not linked to future MACE risk, neither in uni- (HR per log_2_ increase 0.95, 0.53–1.70; *P* = .90) nor multivariable-adjusted analysis (HR 1.48, 0.73–2.99; *P* = .30). However, both hs-CRP and JCAD were strongly linked to 1-year MACE risk in univariable analysis (HR 1.17, 95% CI 1.06–1.30; *P* = .0020; and HR 1.29, 95% CI 1.03–1.62; *P* = .027, respectively) (*Figure 2*). These associations prevailed in multivariable-adjusted analyses, transitioning into a 1.16- and 1.27-fold increase in MACE risk per doubling in hs-CRP [adjusted HR (aHR) 1.16, 1.03–1.30; *P* = .015] and JCAD (aHR 1.27, 1.01–1.60; *P* = .039), respectively, independently of conventional risk factors.

**Figure 3:**
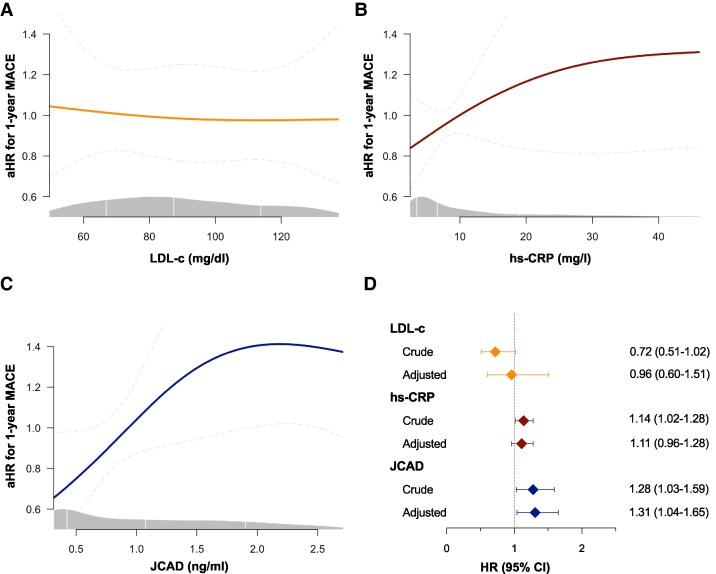
Predictors of 1-year MACE in patients at RIR (on-statin hs-CRP ≥2.0 mg/L): non-linear associations between LDL-c (*A*; yellow), hs-CRP (*B*; red), and JCAD (*C*; blue) and 1-year MACE risk are shown using three-knot restricted cubic spline curves, with knots fixed at the 25th, 50th, and 75th percentiles. Crude and adjusted ratios of the hazard rates for each biomarker (mutually adjusted for each other) are shown in (*D*). Multivariable models include sex, age, JCAD, hs-CRP, and LDL-c, with biomarker data being log_2_-transformed. Note that tilted squares represent HR with line lengths corresponding to 95% confidence intervals. To convert cholesterol levels to millimoles per litre, multiply by 0.0259. aHR, adjusted hazard ratio; HR, hazard ratio; hs-CRP, high-sensitivity C-reactive protein; JCAD, junctional protein associated with coronary artery disease; LDL-c, LDL cholesterol; MACE, major adverse cardiovascular events

Similarly, in those at RIR, LDL-c was not linked to future MACE risk, neither in uni- (HR 0.72, 0.51–1.02; *P* = .062) nor multivariable-adjusted analysis (aHR 0.96, 0.60–1.51; *P* = .80), regardless of LDL-c levels (*Figure 3*). Though a weak association of hs-CRP with MACE risk was noted in univariable analysis (HR 1.14, 1.02–1.28; *P* = .025), this association did not prevail after adjustment of potential confounders (aHR 1.11, 0.96–1.28; *P* = .15). Of interest, however, when compared with those at RLR, the JCAD–MACE association was similarly noted in patients at RIR, with MACE risk being increased by 28% per doubling in JCAD plasma levels in uni- (HR 1.28, 1.03–1.59; *P* = .026) and by 31% in multivariable-adjusted analysis (aHR 1.31, 1.04–1.65; *P* = .022), respectively.

**Figure 4:**
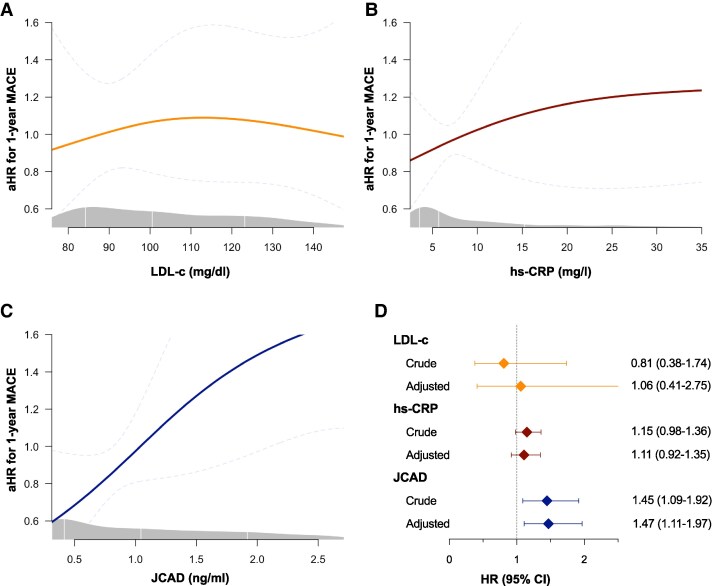
Residual lipid and inflammatory risk [LDL-c ≥70 mg/dL or ≥1.8 mmol/L and hs-CRP ≥2.0 mg/L] and predictors of 1-year MACE: multivariable-adjusted three-knot restricted cubic spline curves (with knots fixed at the 25th, 50th, and 75th percentiles) on the associations between LDL-c (*A*; yellow), hs-CRP (*B*; red), and JCAD (*C*; blue) with MACE risk 1 year after the index ACS are shown. The crude and adjusted HR for LDL-c, hs-CRP, and JCAD (mutually adjusted for each other) are shown in (*D*). Multivariable models include sex, age, JCAD, hs-CRP, and LDL-c. Note that biomarker data were log_2_-transformed. Tilted squares indicate the estimates, with line lengths representing the 95% confidence intervals. To convert cholesterol levels to millimoles per litre, multiply by 0.0259. ACS, acute coronary syndrome; aHR, multivariable-adjusted hazard ratio; CI, confidence interval; HR, hazard ratio; hs-CRP, high-sensitivity C-reactive protein; JCAD, junctional protein associated with coronary artery disease; LDL-c, LDL cholesterol; MACE, major adverse cardiovascular events

In those at RILR, neither LDL-c nor hs-CRP was linked to MACE risk in uni- (HR 0.81, 0.38–1.74; *P* = .60; and 1.15, 0.98–1.36; *P* = .090) or multivariable-adjusted analysis (aHR 1.06, 0.41–2.75; *P* = .99; and 1.11, 0.92–1.35; *P* = .30), irrespectively of plasma LDL-c or hs-CRP levels (*Figure 4*). Notably, however, JCAD retained strong predictive utility also in this high-risk population, transitioning into a 1.45-fold increased MACE risk per doubling in JCAD plasma levels (HR 1.45, 1.09–1.92; *P* = .010). Similar observations were made in multivariable-adjusted analysis accounting for potential confounders, with a doubling in JCAD plasma levels reflecting into a 47% increase in MACE risk at 1 year (HR 1.47, 1.11–1.97; *P* = .0080). In sensitivity analyses, the association between JCAD and 1-year MACE risk was independent of prehospital delay, prior use of antiplatelet therapy or (direct) oral anticoagulants (see Supplementary data online, *Figure S4*). The JCAD–MACE association was confined to patients at RLR, RIR, or RILR but was not observed in control patients not at residual risk (see Supplementary data online, *Figure S5*). When added to a baseline prediction model, none of the biomarkers tested (i.e. JCAD, hs-CRP, LDL-c) resulted in improved discriminatory performance; however, the numerically largest increase in Harrell’s *C* across all residual risk groups was observed when JCAD was included (see Supplementary data online, *Tables S3* and *S4*).

### *Junctional protein associated with coronary artery disease* links to impaired endogenous fibrinolysis and major adverse cardiovascular events

**Figure 5:**
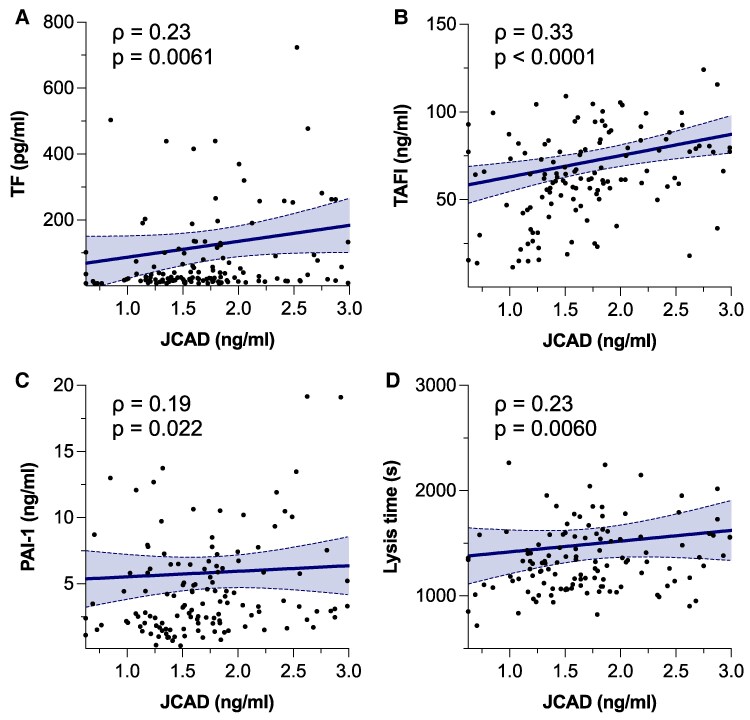
Spearman correlation between JCAD and features of impaired endogenous fibrinolysis. Correlation between JCAD and (*A*) TF, (*B*) TAFI (*C*) PAI-1, and (*D*) baseline lysis time, the latter determined by an established point-of-care global thrombosis test. A simple linear regression and 95% confidence bands of the best fitted line is plotted. JCAD, junctional protein associated with coronary artery disease; PAI-1, plasminogen activator inhibitor-1; TAFI, thrombin activatable fibrinolysis inhibitor; TF, tissue factor

**Figure 6:**
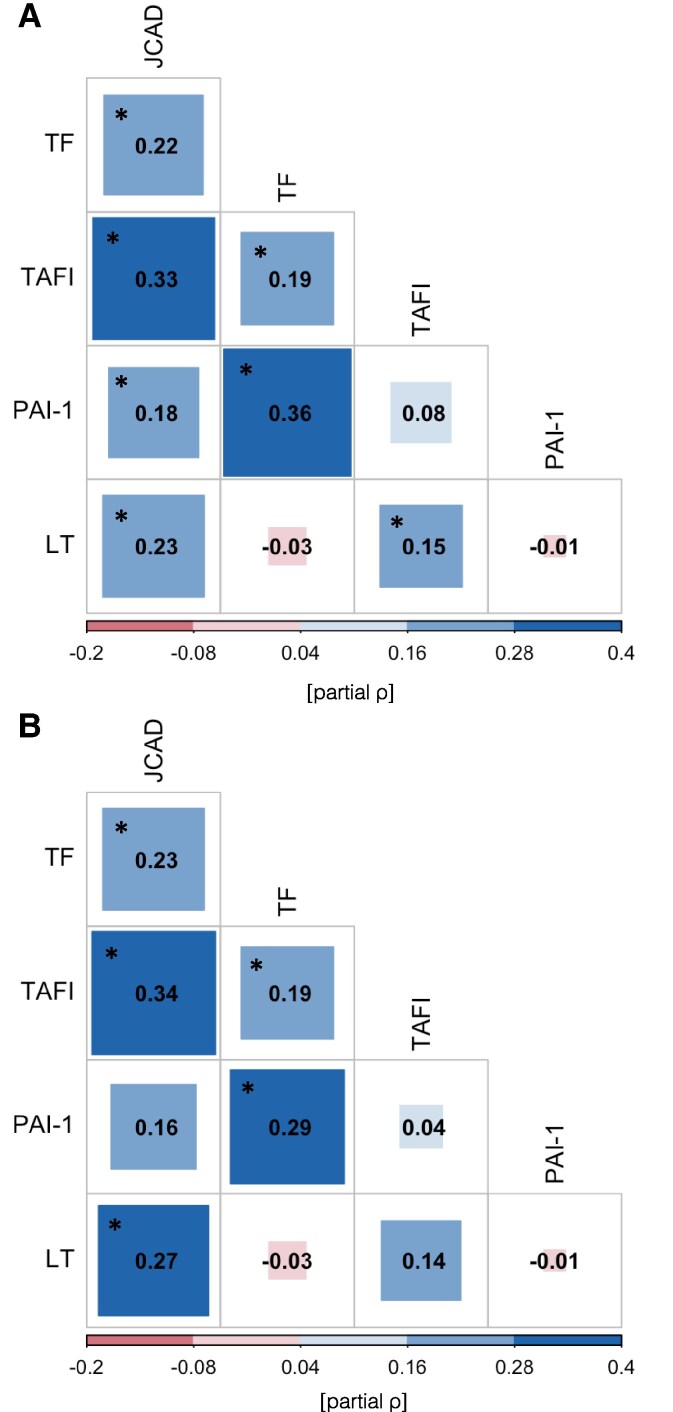
Partial correlation matrix (corrected for eGFR (top) and systemic inflammation (bottom)) showing the independent association of JCAD with proxies of endogenous fibrinolysis. Rank-based pairwise partial correlation coefficients between JCAD, TF, TAFI, and PAI-1, respectively, were calculated while accounting for glomerular filtration rate (*A*), as estimated by the Chronic Kidney Disease Epidemiology Collaboration (CKD-EPI) equation (2009),^44^ or systemic inflammation (*B*), as assessed by CRP. CRP, C-reactive protein; eGFR estimated glomerular filtration rate; JCAD, junctional protein associated with coronary artery disease; PAI-1, plasminogen activator inhibitor-1; TAFI, thrombin activatable fibrinolysis inhibitor; TF, tissue factor. Square areas correspond to the absolute value of partial Spearman correlation coefficients. *P < .05

**Figure 7:**
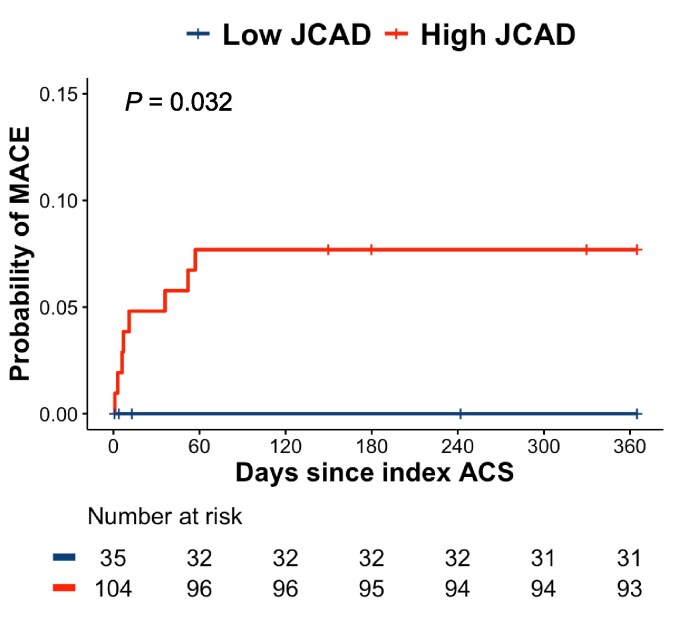
Plasma levels of JCAD and risk of 1-year MACE in RISK-PPCI study participants. Low JCAD refers to <25th percentile. ACS, acute coronary syndrome; JCAD, junctional protein associated with coronary artery disease; MACE, major adverse cardiovascular events

The *junctional protein associated with coronary artery disease* (JCAD) is causally involved in atherosclerosis,^45,46,47^ driving atherothrombotic events predominantly by modulating coagulation and fibrinolysis.^25^ In ACS patients recruited in the RISK-PPCI study undergoing the automated point-of-care GTT (patient characteristics are provided in the Supplementary data online, *Table S5*), high JCAD plasma levels showed a monotonic relationship with accentuated TF (*ρ* = 0.23, *P* = .0061), TAFI (*ρ* = 0.33, *P* < .0001) and PAI-1 plasma levels (*ρ* = 0.19, *P* = .022) (*Figure 5*). Aligning with the above, JCAD correlated positively with baseline lysis time (*ρ* = 0.23, *P* = .0060), an important determinant of ischaemic risk.^10^ The associations between JCAD and TF, TAFI, and lysis time were independent of renal function, as estimated by glomerular filtration rate, and systemic inflammation, as assessed by CRP (*Figure 6*). However, in linear regression analysis adjusting for eGFR and/or CRP, only TF, TAFI and lysis time correlated linearly with JCAD (Supplementary data online, *Table S6*). Similar to the data obtained in SPUM-ACS, high JCAD levels translated into an increased risk of MACE 1 year after the index event in patients undergoing primary PCI (*P* = .032) (*Figure 7*; Supplementary data online, *Figure S6*).

## Discussion

Harnessing two independent prospective ACS cohorts from two different countries, we show that (i) ACS patients at RLR, RIR, or both (RILR), remain at high ischaemic risk, (ii) plasma levels of JCAD, but not LDL-c or hs-CRP, associate consistently with MACE risk irrespectively of type of residual risk, and (iii) high circulating JCAD independently links to higher levels of pro-thrombotic mediators, impaired endogenous fibrinolysis and MACE in prospectively recruited patients with ACS (*Structured Graphical Abstract*).

Of note, patients at RLR, defined as on-statin LDL-c ≥70 mg/dL (≥1.80 mmol/L), exhibited 55% higher risk of MACE risk when compared with PS-matched controls. Similarly, those at RIR (hs-CRP ≥2.0 mg/L) as well as those at RILR (both LDL-c ≥70 mg/dL and hs-CRP ≥2.0 mg/L) had 1.8- and 1.75-fold higher hazards of 1-year MACE, respectively, relative to control patients. Statins reduce levels of both hs-CRP and LDL-c which is associated with lower MACE risk, but both biomarkers contribute independently to overall ischaemic risk.^48,49,50^ Aggressive LDL-c lowering leads to a relative risk reduction in MACE (with each 1.0 mmol/L reduction corresponding to an annual MACE risk reduction of ∼20%),^51^ but ∼1 out of 20 stabilized patients achieving a median LDL-c <50 mg/dL (<1.4 mmol/L) still experiences MACE during 1 year of follow-up.^15,16^ The lack of predictive value of hs-CRP in patients at RIR in the present study contrasts with findings from major trials such as PROMINENT, REDUCE-IT, and STRENGTH.^52–55^ This discrepancy may be due to differences in study design, distinct patient populations (real-world data vs selected trial populations), different in-/exclusion criteria, and post-ACS settings. These differences highlight the challenges of translating trial findings to broader clinical settings. Indeed, even in patients achieving currently recommended LDL-c targets, residual cardiovascular risk remains substantial, with targeted anti-inflammatory agents, including interleukin-6 inhibitors (e.g. ziltivekimab), being currently under evaluation.^56^ In the combined residual risk group (RILR), neither LDL-c nor hs-CRP were independently associated with MACE, and the incidence of events was slightly lower than in the RIR group. This may reflect population heterogeneity, as well as the distinct biological timelines of risk modulation: while inflammation may exert short-term effects on event risk, the benefits of LDL-c lowering typically accumulate over longer periods, as shown in ODYSSEY OUTCOMES, FOURIER, and 4S trials.^12,15,16^ The 1-year follow-up may therefore have favoured the detection of inflammatory over lipid-mediated effects. Additionally, the use of dichotomized baseline cut-offs for risk definition may limit the ability to capture complex interactions between these pathways. Collectively, these findings highlight the currently unmet need for novel targets to further reduce residual risk, particularly in those exceeding guideline-recommended LDL-c thresholds despite optimal medical therapy.^2,47^

Besides residual lipid and inflammatory risk, other key contributors to residual risk in patients with established atherosclerotic cardiovascular disease include triglycerides, lipoprotein(a), diabetes, and thrombotic risk.^11^ The latter stands out as no single biomarker of residual thrombotic risk is recommended by guidelines, and novel targets remain to be identified to adequately balance ischaemic vs bleeding risks.^9^ While several biomarkers of thrombotic risk have been evaluated—including TF, PAI-1, D-dimer, soluble urokinase-type plasminogen activator receptor, and platelet-derived microparticles—none are currently established for clinical use to guide personalized antithrombotic therapy.^57–61^ Among these, JCAD holds promise as a superior biomarker of thrombotic residual risk given its dual mechanistic involvement in atherosclerosis and thrombosis, as well as its consistent association with prothrombotic pathways and ischaemic outcomes in ACS patients at high residual risk.

Indeed, our results support the notion that JCAD plasma levels associate independently with increased MACE risk in ACS patients at RLR, RIR, or both (RILR) beyond LDL-c and hs-CRP. Initially identified by GWAS, JCAD has been causally implicated in atherosclerosis, vascular inflammation, and arterial thrombosis in experimental studies.^23,24,25,45,46,62^ While JCAD promotes early stages of atherosclerosis through the Hippo signalling pathway,^62^ its effects on thrombosis largely depend on the phosphoinositide 3-kinases/Akt pathway.^25^ While evidence on the pro-atherogenic role of intracellular JCAD is growing,^23–45^ the pathophysiological role of its extracellular and thus circulating form remains to be investigated.^47^ In the present study, JCAD plasma levels were independently linked to an increased MACE risk irrespective of residual risk type. Notably, in RISK-PPCI study participants, JCAD correlated well with prothrombotic factors, including TF, TAFI, PAI-1, and lysis time, the latter representing a potent determinant of ischaemic risk.^10^ This observation aligns with our prior work showing that JCAD promotes arterial thrombus formation in mice, with TF and PAI-1 expression being blunted in JCAD-deprived endothelial cells.^25^ Several markers of thrombotic risk, including on-treatment platelet reactivity, as assessed by the VerifyNow® assay, and platelet FcγRIIa, are linked to heightened ischaemic risk in patients at high residual risk.^63,64^ Thus far, however, platelet-derived biomarkers failed to enter clinical practice. This might be due to several factors, including the notion that ischaemic risk is not only determined by platelet function, but a complex interplay of lipids, inflammation, and cardiometabolic risk factors.^9^ In this regard, JCAD may represent an unique class of biomarker, as it is causally involved in atherothrombosis,^23–25,45,46,62^ reflecting upstream endothelial dysfunction rather than isolated platelet (dys-)function and reactivity. Indeed, in our experimental work, siRNA-mediated JCAD knockdown resulted in improved outcomes in models of both arterial thrombosis and stroke.^25,35^ While additional mechanistic studies are warranted, these findings suggest that JCAD might not only serve as a risk marker but also a potential therapeutic target. Considering that residual risk is increasingly recognized as a multifaceted process involving lipid, inflammatory, and thrombotic pathways,^9,47^ comprehensive risk reduction may require a combination of therapies targeting these pathways. While novel agents, including emerging lipid-lowering drugs, glucagon-like peptide-1 receptor agonists, and sodium-glucose co-transporter 2 (SGLT2) inhibitors have changed the management of many cardiometabolic conditions,^65–67^ both the DAPA-MI and EMPACT-MI trials failed to show a benefit of SGLT2 inhibition on hard cardiovascular outcomes in patients with ACS.^68,69^ The optimal duration and intensity of dual antiplatelet therapy remain subjects of ongoing debate, and current guidelines increasingly advocate for personalized approaches balancing individual thrombotic and bleeding risks, with JCAD emerging as a promising biomarker for contemporary risk stratification. While, at present, no therapeutic strategy exists to target JCAD, well-designed studies are warranted to explore whether JCAD modulation can effectively reduce residual cardiovascular risk on the background of contemporary management strategies.

### Strengths and limitations

SPUM-ACS is among the largest prospectively designed multicentre ACS cohorts globally with granular phenotyping of recruited patients and independent event adjudication by an expert committee comprising three board certified cardiologists blinded to baseline characteristics using pre-specified adjudication forms.^26,29–31^ Moreover, biomarker measurements (i.e. hs-CRP, JCAD) were done centrally,^25,26,35^ with LDL-c levels derived from the well-validated Sampson equation,^28,36^ assuring high data quality. However, potential limitations of this study warrant discussion. First, though the SPUM-ACS cohort is among the best characterized and largest ACS cohorts worldwide, relatively few patients were at RLR, RIR, or both (RILR); thus, a potential selection bias cannot be excluded. However, JCAD–MACE associations were similarly observed across different subgroups of residual risk, strongly arguing against such a systematic error. To avoid model overfit, we refrained from performing additional subgroup analyses in SPUM-ACS stratified by ACS type. Given differences in the pathophysiology of STEMI vs non-ST-elevation (NSTE)-ACS,^1^ future studies would need to assess whether the JCAD–MACE associations are similarly observed in patients with STEMI vs NSTE-ACS. Second, residual risk groups in our study were defined based on lipid and inflammatory markers measured during the index hospitalization, which may be influenced by acute-phase responses, including stress-induced fluctuations in LDL-c and elevations in hs-CRP due to myocardial injury. As such, these measurements might not fully reflect steady-state post-treatment levels, possibly favouring distinct risk stratification. However, our goal was to evaluate the prognostic value of JCAD in real-world ACS patients shortly after presentation—when treatment decisions have to be made. Future studies with serial biomarker measurements post-discharge may help distinguish acute from persistent residual risk and clarify potential interactions between biomarker dynamics and JCAD-associated MACE risk. Additionally, the herein used LDL-c thresholds of 70 mg/dL (≥1.8 mmol/L) were based on earlier guideline definitions applicable during the recruitment period; this may limit generalizability to contemporary populations where more stringent LDL-c targets (<55 or <70 mg/dL) are recommended, potentially attenuating the relative impact of additional biomarkers such as JCAD. Third, GTT-derived lysis time data, which reflect procoagulant or impaired fibrinolytic activity, were unavailable in SPUM-ACS, limiting mechanistic insight into thrombotic risk pathways in this cohort. Indeed, the present study is subject to any limitation inherent to its design, including these methodological limitations as well as residual confounding. To minimize a potential confounding effect while avoiding model overfit in the setting of marked covariate imbalance,^70,71^ PS matching was done, with the PS being derived from *a priori*-defined covariates related to both group assignment and ischaemic outcomes. To mitigate potential missing data bias, PS matching was performed on multiply imputed data (*n* = 20). Finally, the present investigation was done within cohorts mainly comprising Caucasian patients (SPUM-ACS; RISK-PPCI study) which may limit the generalizability of the findings to broader, more diverse populations with different genetic backgrounds and environmental risk factors.

## Conclusions

In aggregate, our findings reinforce the urgent need for more aggressive secondary prevention strategies in patients with a recent ACS, particularly in those with residual lipid and/or inflammatory risk, using novel therapeutic strategies. While intensifying of LDL-c lowering therapy, combined with targeted anti-inflammatory approaches, may mitigate ischaemic risk in this high-risk population to some degree, novel targets beyond lipids and inflammation deserve focus. In this regard, given its mechanistic role in endothelial dysfunction, atherosclerosis, and arterial thrombosis,^23–25,45^ coupled with its consistent associations with MACE risk across residual risk types, JCAD represents a promising candidate and potential therapeutic target to lower the burden of RLR, RIR, and RILR in patients with a recent ACS. Additional studies are warranted to explore whether JCAD modulation can effectively reduce ischaemic risk in these high-risk patients, irrespective of ACS type and presence or absence of residual risk phenotypes.

## Supplementary Material

ehaf979_Supplementary_Data

## Data Availability

Due to strict data protection regulations, the authors do not have authorization to provide unrestricted data access. Data requests from qualified investigators can be made to the corresponding authors and will be considered by the SPUM-ACS and RISK-PPCI steering committees, subject to institutional and ethical committee approvals.

## Pre-registered Clinical Trial Number

The pre-registered clinical trial numbers are NCT01000701 and NCT02562690.
