# Supplementary material for: The junctional protein associated with coronary artery disease predicts adverse cardiovascular events in patients with acute coronary syndromes at high residual risk
Source: Eur Heart J. 2025 Dec 23;47(28):3693–707. doi: 10.1093/eurheartj/ehaf979 (PMC13384729; doi:10.1093/eurheartj/ehaf979)
Supplement: ehaf979_Supplementary_Data [file ehaf979_supplementary_data.docx]

**Supplementary material**

**The junctional protein JCAD predicts adverse cardiovascular events in patients with acute coronary syndromes at high residual risk**

**Simon Kraler, Luca Liberale, Amedeo Tirandi, Margherita Moriero, Yifan Wang, Mohamed Farag, Federico Carbone, Maria B. Bertolotto, Valentina Pusterla, Davide Ramoni, Stefano Ministrini, Yustina M. Puspitasari, Francesco Bruno, Lorenz Räber, Davide Di Vece, Christian Templin, Olivier Muller, François Mach, Filippo Crea, Giovanni G. Camici, Tetiana Lapikova-Bryhinska, Alexander Akhmedov, Arnold von Eckardstein, Diana A. Gorog, Fabrizio Montecucco and Thomas F. Lüscher on behalf of the SPUM-ACS and RISK-PPCI investigators**

**Table of contents**

**Figure S1** Study flow-chart

**Figure S2** Venn diagram showing patient subgroups and numbers

**Figure S3** Risk of MACE among patients with residual risk compared to the full cohort

**Figure S4** Multivariable-adjusted biomarker-based prediction of 1-year MACE risk across residual risk subtypes accounting for pre-hospital delays or SAPT and/or anticoagulation use

**Figure S5** Uni- and multivariable-adjusted association of JCAD biomarker levels and 1-year MACE risk in patients not at residual risk

**Figure S6** Association of continuous JCAD and adverse cardiovascular events in RISK-PPCI study participants

**Table S1** Degree of missing data in SPUM-ACS

**Table S2** Uni- and multivariable-adjusted risk of MACE among patients with residual risk compared to the full cohort of patients not assigned to RLR, RIR, or RILR groups

**Table S3** Discriminatory performance of biomarker-enhanced risk prediction models for 1-year MACE across residual risk groups

**Table S4** Prediction error of biomarker-enhanced risk prediction models for 1-year MACE across residual risk groups

**Table S5** Characteristics of RISK-PPCI study participants with available plasma samples

**Table S6** Linear associations of JCAD plasma levels with prothrombotic markers

**Figure S1.** Study flow-chart.**
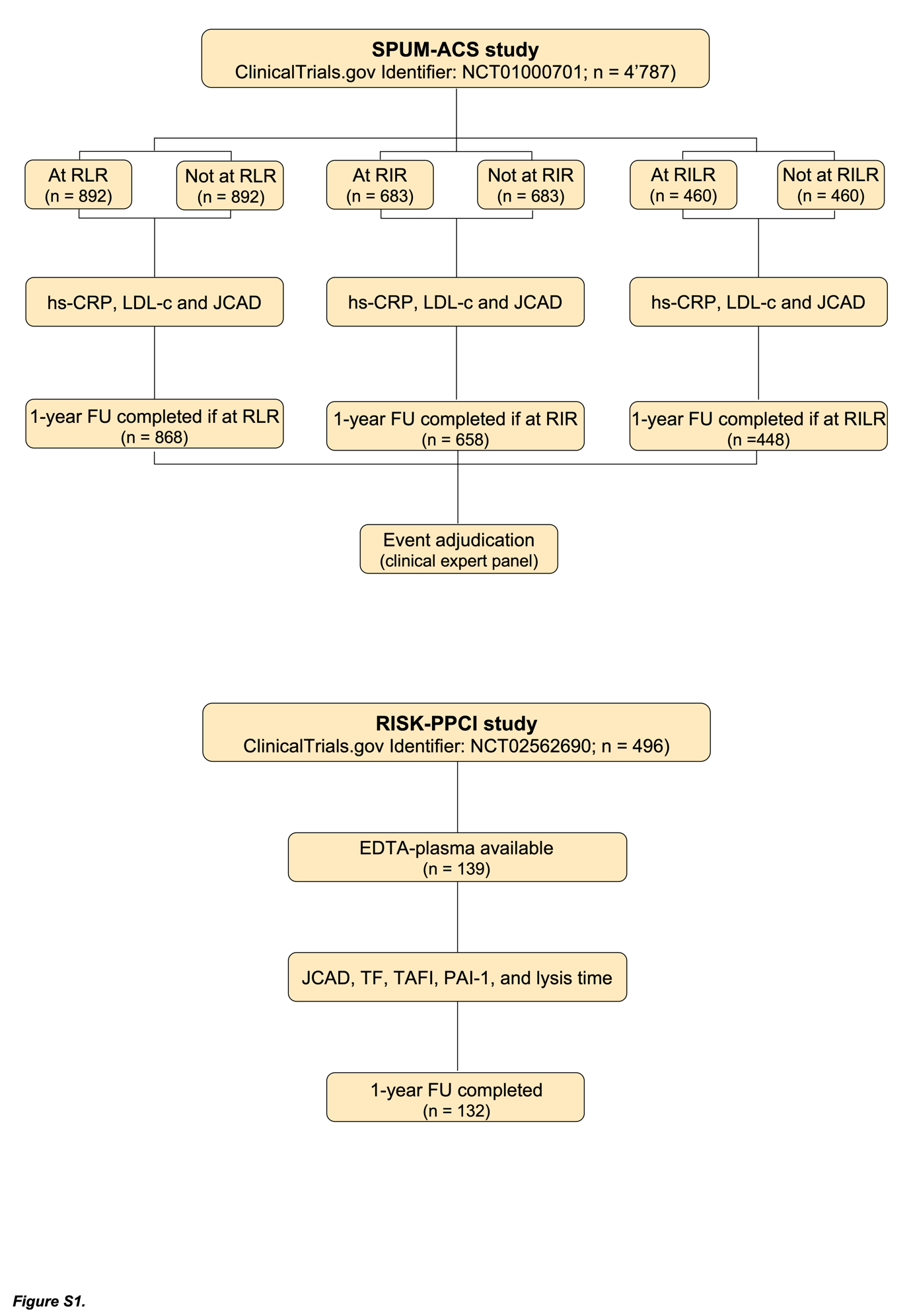
**

FU denotes follow-up, hs-CRP high-sensitivity C-reactive protein, JCAD Junctional Protein Associated with Coronary Artery Disease, LDL-c low-density lipoprotein cholesterol, PAI-1 plasminogen activator inhibitor-1, RIR residual inflammatory risk, RLR residual lipid risk, and RILR residual inflammatory and lipid risk, TAFI thrombin activatable fibrinolysis inhibitor, and TF tissue factor.

**
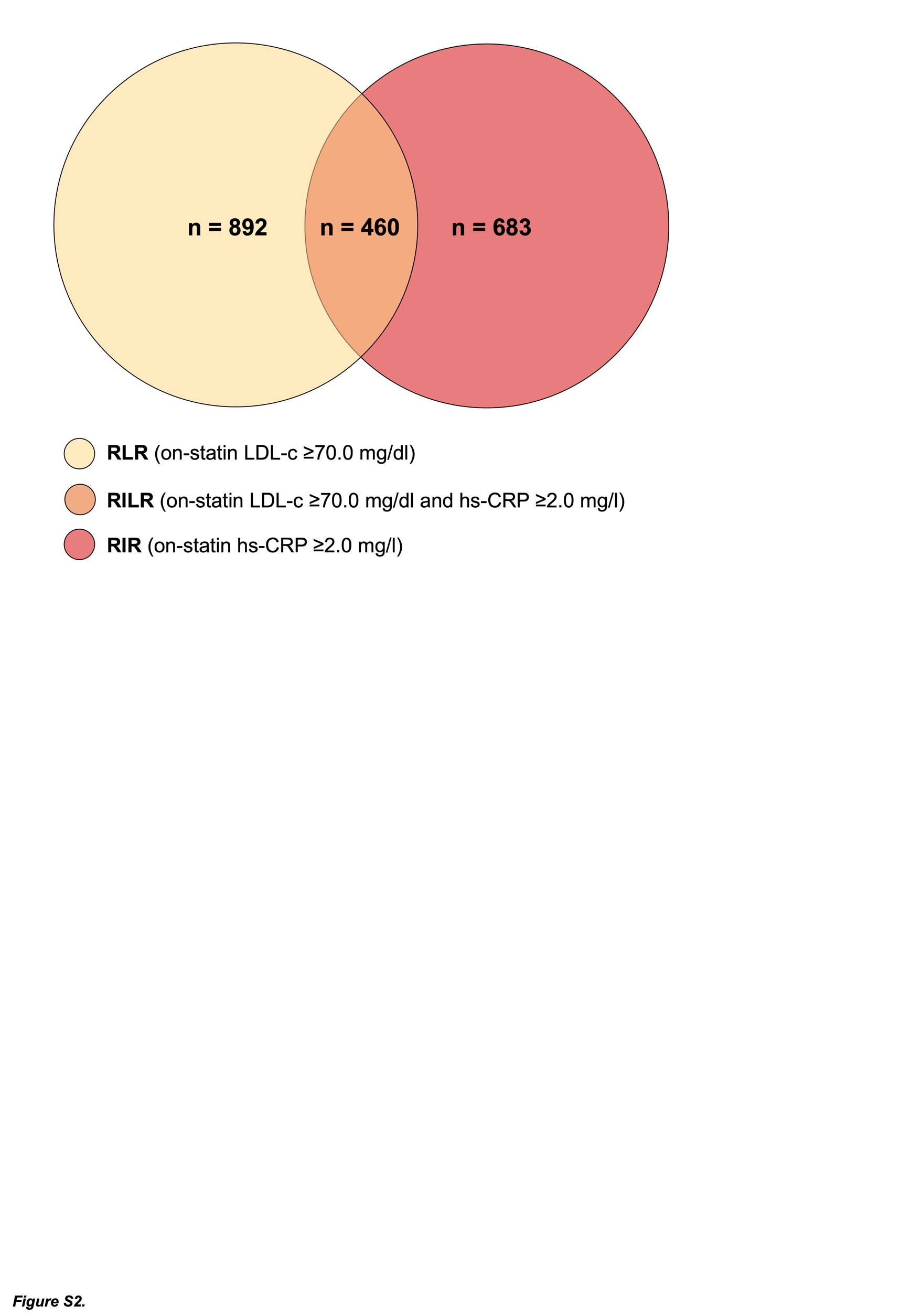
Figure S2.** Venn diagram showing patient subgroups and numbers in SPUM-ACS.

Hs-CRP denotes high-sensitivity C-reactive protein, LDL-c low-density lipoprotein cholesterol, RIR residual inflammatory risk, RLR residual lipid risk, and RILR residual inflammatory and lipid risk. To convert cholesterol levels to millimoles per litre, multiply by 0.0259.

**Figure S3**. Risk of MACE among patients with residual risk compared to the full cohort.

(**A**) RLR refers to residual lipid risk (on-statin LDL-c ≥70 mg/dL). (**B**) RIR refers to residual inflammatory risk (on-statin hs-CRP ≥2 mg/L). (**C**) RILR refers to combined residual inflammatory and lipid risk (LDL-c ≥70 mg/dL and hs-CRP ≥2 mg/L). Right-censored observations are indicated by tick marks. Cumulative 1-year incidence of MACE was 6.70% (95% CI 5.89 – 7.51) for controls and 8.67% (95% CI 6.80 – 10.51) for patients at RLR, 12.27% (95% CI 9.76 – 14.71) for patients at RIR, and 10.72% (95% CI 7.84 – 13.52) for patients at RILR. Cumulative 1-year MACE incidence and HR with their corresponding 95% CI were calculated by the Kaplan-Meier method and Cox proportional hazard regression models, respectively. To convert cholesterol levels to millimoles per litre, multiply by 0.0259. CI denotes confidence interval, HR hazard ratio, MACE major adverse cardiovascular events, RIR residual inflammatory risk, RLR residual lipid risk, RILR residual inflammatory and lipid risk.


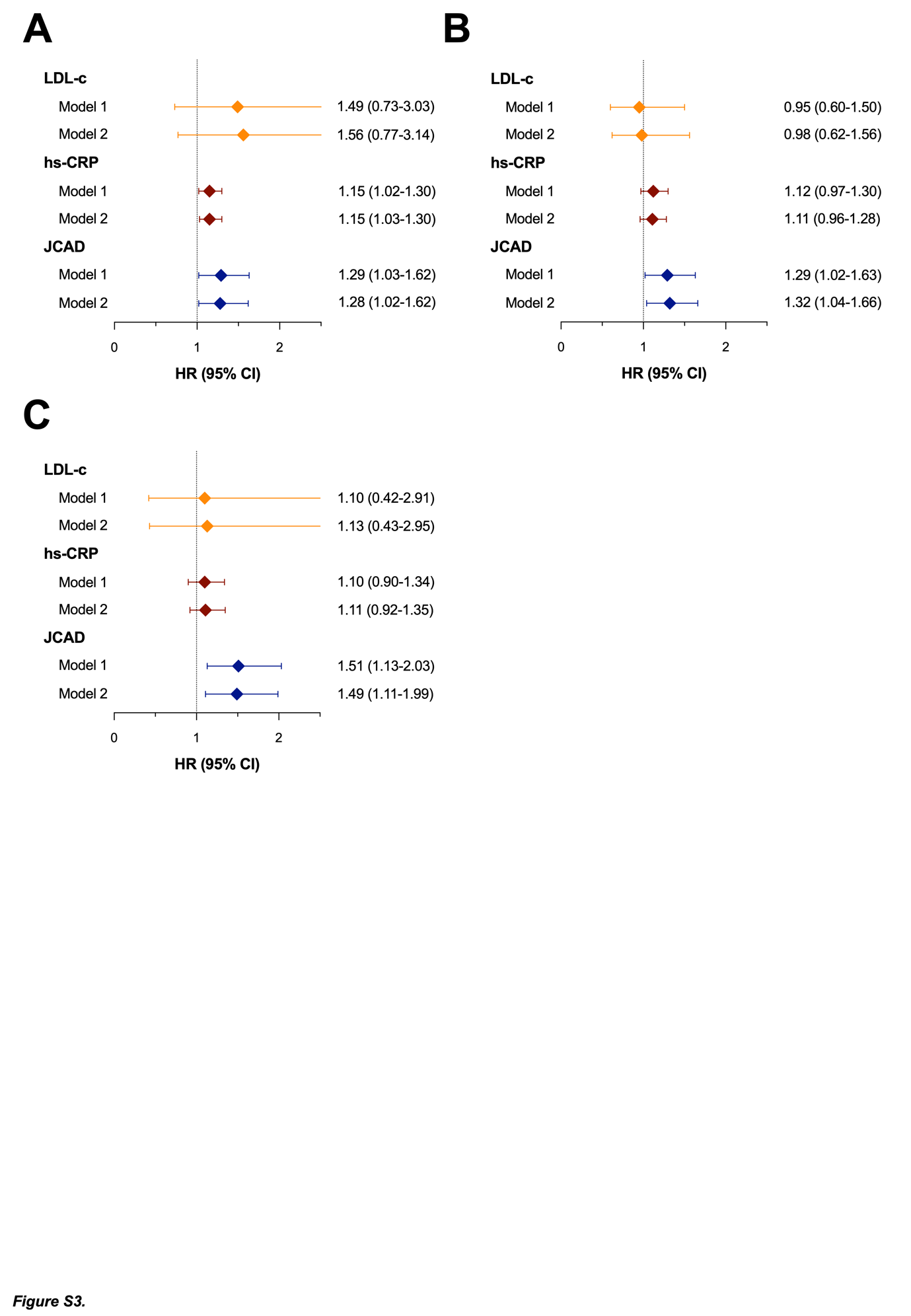
**Figure S4.** Multivariable-adjusted biomarker-based prediction of 1-year MACE risk across residual risk subtypes accounting for pre-hospital delays (model 1) or SAPT and/or anticoagulation use (model 2).

Independent associations between LDL-c (**yellow**), hs-CRP (**red**), and JCAD (**blue**) levels and 1-year risk of major adverse cardiovascular events (MACE) are shown in patients stratified by residual lipid risk (RLR; panel **A**), residual inflammatory risk (RIR; panel **B**), and combined residual inflammatory and lipid risk (RILR; panel **C**). Model 1 includes sex, age, JCAD, hs-CRP, LDL-c, and onset-to-presentation time. Model 2 includes sex, age, JCAD, hs-CRP, LDL-c, (D)OAC use, and presence of SAPT. Biomarker data were log2-transformed. Hazard ratios of models 1 and 2 are depicted using tilted squares with line lengths corresponding to 95% CIs. CI denotes confidence interval, (D)OAC (direct) oral anticoagulants, HR hazard ratio, hs-CRP high-sensitivity C-reactive protein, JCAD Junctional Protein Associated with Coronary Artery Disease, LDL-c low-density lipoprotein cholesterol, and SAPT single antiplatelet therapy.

**Figure S5.** Uni- and multivariable-adjusted association of JCAD biomarker levels and 1-year MACE risk in patients not at residual risk.

Control patients refer to PS-matched controls not found to be at residual lipid risk (RLR), residual inflammatory risk (RIR), or combined risk (RILR), as defined in *Figure S1*. JCAD biomarker data were log2-transformed. Hazard ratios are depicted using tilted squares with line lengths corresponding to 95% CIs. The multivariable-adjusted models include sex, age, JCAD, hs-CRP, and LDL-c as independent variables. CI denotes confidence interval, HR hazard ratio, JCAD Junctional Protein Associated with Coronary Artery Disease, and PS propensity score.

**Figure S6**. Association of continuous JCAD and adverse cardiovascular events in RISK-PPCI study participants.

Multivariable-adjusted 3-knot restricted cubic spline curves (with knots fixed at the 25th, 50^th^, and 75th percentiles) showing the associations between JCAD and 1-year MACE risk in RISK-PPCI study participants. To avoid model overfit, independent variables were restricted to sex, age, and JCAD. aHR denotes multivariable-adjusted hazard ratio, JCAD Junctional Protein Associated with Coronary Artery Disease, and MACE major adverse cardiovascular events.

**Table S1**. Degree of missing data in SPUM-ACS.

| **Patient cohort** | **Variable** | **N valid** | **N missing (%)** |
| --- | --- | --- | --- |
| **RLR** | Age | 892 | 0 (0%) |
|  | Sex | 892 | 0 (0%) |
|  | GRACE risk | 892 | 0 (0%) |
|  | Anterior MI | 892 | 0 (0%) |
|  | LVEF | 536 | 356 (39.9%) |
|  | SBP (mmHg) | 884 | 8 (0.9%) |
|  | BMI (kg/m^2^) | 877 | 15 (1.7%) |
|  | BSA (m^2^) | 877 | 15 (1.7%) |
|  | Hx of smoking | 876 | 16 (1.8%) |
|  | Drinks per day | 784 | 108 (12.1%) |
|  | eGFR (mL/min/1.73 m^2^) | 878 | 14 (1.6%) |
|  | H_x_ of DM | 892 | 0 (0%) |
|  | FH_x_ of CAD | 882 | 10 (1.1%) |
|  | H_x_ of PAD | 892 | 0 (0%) |
|  | H_x_ of stroke/TIA | 892 | 0 (0%) |
|  | H_x_ of HF | 892 | 0 (0%) |
|  | H_x_ of dialysis | 892 | 0 (0%) |
|  | H_x_ of malignancy | 891 | 1 (0.1%) |
|  | hs-CRP (mg/L) | 822 | 70 (7.8%)* |
|  | NT-proBNP (ng/L) | 821 | 71 (8%)* |
|  | hs-cTnT (ng/L) | 824 | 68 (7.6%)* |
|  | Hb (g/dL) | 892 | 0 (0%) |
|  | HDL-c (mg/dL) | 892 | 0 (0%) |
|  | Triglycerides (mg/dL) | 892 | 0 (0%) |
|  | LDL-c ≥70 mg/dL | 892 | 0 (0%) |
|  | JCAD (pg/mL) | 808 | 84 (9.4%)* |
| **RIR** | Age | 683 | 0 (0%) |
|  | Sex | 683 | 0 (0%) |
|  | GRACE risk | 683 | 0 (0%) |
|  | Anterior MI | 683 | 0 (0%) |
|  | LVEF | 407 | 276 (40.4%) |
|  | SBP (mmHg) | 675 | 8 (1.2%) |
|  | BMI (kg/m^2^) | 672 | 11 (1.6%) |
|  | BSA (m^2^) | 672 | 11 (1.6%) |
|  | Hx of smoking | 666 | 17 (2.5%) |
|  | Drinks per day | 587 | 96 (14.1%) |
|  | eGFR (mL/min/1.73 m^2^) | 671 | 12 (1.8%) |
|  | H_x_ of DM | 683 | 0 (0%) |
|  | FH_x_ of CAD | 679 | 4 (0.6%) |
|  | H_x_ of PAD | 683 | 0 (0%) |
|  | H_x_ of stroke/TIA | 683 | 0 (0%) |
|  | H_x_ of HF | 682 | 1 (0.1%) |
|  | H_x_ of dialysis | 683 | 0 (0%) |
|  | H_x_ of malignancy | 682 | 1 (0.1%) |
|  | hs-CRP (mg/L) | 683 | 0 (0%)* |
|  | NT-proBNP (ng/L) | 680 | 3 (0.4%)* |
|  | hs-cTnT (ng/L) | 683 | 0 (0%)* |
|  | Hb (g/dL) | 683 | 0 (0%) |
|  | HDL-c (mg/dL) | 658 | 25 (3.7%) |
|  | Triglycerides (mg/dL) | 660 | 23 (3.4%) |
|  | LDL-c ≥70 mg/dL | 656 | 27 (4%) |
|  | JCAD (pg/mL) | 626 | 57 (8.3%)* |
| **RILR** | Age | 460 | 0 (0%) |
|  | Sex | 460 | 0 (0%) |
|  | GRACE risk | 460 | 0 (0%) |
|  | Anterior MI | 460 | 0 (0%) |
|  | LVEF | 278 | 182 (39.6%) |
|  | SBP (mmHg) | 455 | 5 (1.1%) |
|  | BMI (kg/m^2^) | 453 | 7 (1.5%) |
|  | BSA (m^2^) | 453 | 7 (1.5%) |
|  | Hx of smoking | 450 | 10 (2.2%) |
|  | Drinks per day | 397 | 63 (13.7%) |
|  | eGFR (mL/min/1.73 m^2^) | 450 | 10 (2.2%) |
|  | H_x_ of DM | 460 | 0 (0%) |
|  | FH_x_ of CAD | 458 | 2 (0.4%) |
|  | H_x_ of PAD | 460 | 0 (0%) |
|  | H_x_ of stroke/TIA | 460 | 0 (0%) |
|  | H_x_ of HF | 460 | 0 (0%) |
|  | H_x_ of dialysis | 460 | 0 (0%) |
|  | H_x_ of malignancy | 459 | 1 (0.2%) |
|  | hs-CRP (mg/L) | 460 | 0 (0%)* |
|  | NT-proBNP (ng/L) | 458 | 2 (0.4%)* |
|  | hs-cTnT (ng/L) | 460 | 0 (0%)* |
|  | Hb (g/dl) | 460 | 0 (0%) |
|  | HDL-c (mg/dL) | 460 | 0 (0%) |
|  | Triglycerides (mg/dL) | 460 | 0 (0%) |
|  | LDL-c ≥70 mg/dL | 460 | 0 (0%) |
|  | JCAD (pg/mL) | 423 | 37 (8%)* |

BMI denotes body mass index, BSA body surface area, CAD coronary artery disease, DM diabetes mellitus, eGFR estimated glomerular filtration rate, FHx family history, GRACE Global Registry of Acute Coronary Events, Hb haemoglobin, HDL high-density lipoprotein cholesterol, HF heart failure, hs-CRP high-sensitivity C-reactive protein, hs-cTnT high-sensitivity cardiac troponin-T, Hx history, JCAD Junctional Protein Associated with Coronary Artery Disease, LDL-c low-density lipoprotein cholesterol, LVEF left-ventricular ejection fraction, MI myocardial infarction, NT-proBNP N-terminal pro–B-type natriuretic peptide, PAD peripheral artery disease, RIR residual inflammatory risk, RILR residual inflammatory and lipid risk, RLR residual lipid risk, SBP systolic blood pressure, TIA transient ischemic attack. *Biomarker data were unavailable if patient sample was missing or grossly haemolytic. To convert cholesterol levels to millimoles per litre, multiply by 0.0259.

**Table S2**. Uni- and multivariable-adjusted risk of MACE among patients with residual risk compared to the full cohort of patients not assigned to RLR, RIR, or RILR groups.

|  | **RLR** | | **RIR** | | **RILR** | | **Not at residual risk** |
| --- | --- | --- | --- | --- | --- | --- | --- |
|  | **HR** | **95% CI** | **HR** | **95% CI** | **HR** | **95% CI** | *reference* |
| Model 1 | 1.30 | 1.00–1.68 | 1.87 | 1.45-2.40 | 1.61 | 1.18-2.19 |  |
| Model 2 | 1.44 | 1.09- 1.89 | 2.00 | 1.53-2.60 | 1.75 | 1.27-2.42 |  |
| Model 3 | 1.54 | 1.17-2.04 | 1.99 | 1.53-2.59 | 1.77 | 1.28-2.45 |  |
| Model 4 | 1.46 | 1.11-1.93 | 1.53 | 1.16-2.02 | 1.40 | 1.01-1.95 |  |
| Model 5 | 1.42 | 1.08-1.89 | 1.52 | 1.19-2.01 | 1.38 | 1.00-1.93 |  |

Model 1 refers to the univariable model. Model 2 includes sex, history of hypercholesterolaemia, smoking history, and a history of heart failure as independent variables. Model 3 includes sex, history of hypercholesterolaemia, smoking history, a history of heart failure, and levels of high-sensitivity cardiac troponin as independent variables. Model 4 includes sex, history of hypercholesterolaemia, smoking history, a history of heart failure, levels of high-sensitivity cardiac troponin, and levels of high-sensitivity C-reactive protein as independent variables. Model 5 includes sex, history of hypercholesterolaemia, smoking history, a history of heart failure, levels of high-sensitivity cardiac troponin, levels of high-sensitivity C-reactive protein, and GRACE risk estimates as independent variables. Patients not at residual risk served as controls. CI denotes confidence interval, HR hazard ratio, MACE major adverse cardiovascular events, RIR residual inflammatory risk, RLR residual lipid risk, and RILR residual inflammatory and lipid risk.

**Table S3**. Discriminatory performance of biomarker-enhanced risk prediction models for 1-year MACE across residual risk groups.

|  | **Model** | **Harrell’s C (new model)** | **Harrell’s C (bsl model)** | **ΔC** | ***P* value*** |
| --- | --- | --- | --- | --- | --- |
| **RLR** | **Individual biomarker models** |  |  |  |  |
|  | Bsl + JCAD | 0.6834 | 0.6642 | 0.0192 | 0.37 |
|  | Bsl + hs-CRP | 0.6775 | 0.6642 | 0.0132 | 0.41 |
|  | Bsl + LDL-c | 0.6678 | 0.6642 | 0.0035 | 0.48 |
|  | **Combined models** |  |  |  |  |
|  | Bsl + JCAD + hs-CRP + LDL-c | 0.6950 | 0.6642 | 0.0308 | 0.30 |
|  | Bsl + hs-CRP + LDL-c | 0.6861 | 0.6642 | 0.0219 | 0.36 |
| **RIR** | **Individual biomarker models** |  |  |  |  |
|  | Bsl + JCAD | 0.7042 | 0.6858 | 0.0183 | 0.37 |
|  | Bsl + hs-CRP | 0.6972 | 0.6858 | 0.0114 | 0.42 |
|  | Bsl + LDL-c | 0.6819 | 0.6858 | -0.0039 | 0.53 |
|  | **Combined models** |  |  |  |  |
|  | Bsl + JCAD + hs-CRP + LDL-c | 0.7082 | 0.6858 | 0.0224 | 0.34 |
|  | Bsl + hs-CRP + LDL-c | 0.6938 | 0.6858 | 0.0080 | 0.44 |
| **RILR** | **Individual biomarker models** |  |  |  |  |
|  | Bsl + JCAD | 0.7383 | 0.7200 | 0.0183 | 0.39 |
|  | Bsl + hs-CRP | 0.7254 | 0.7200 | 0.0054 | 0.47 |
|  | Bsl + LDL-c | 0.7225 | 0.7200 | 0.0026 | 0.48 |
|  | **Combined models** |  |  |  |  |
|  | Bsl + JCAD + hs-CRP + LDL-c | 0.7429 | 0.7200 | 0.0229 | 0.36 |
|  | Bsl + hs-CRP + LDL-c | 0.7237 | 0.7200 | 0.0037 | 0.48 |

Bsl denotes baseline, HR hazard ratio, hs-CRP high-sensitivity C-reactive protein, GRACE Global Registry of Acute Coronary Events, JCAD Junctional Protein Associated with Coronary Artery Disease, LDL-c low-density lipoprotein cholesterol, MACE major adverse cardiovascular events, RIR residual inflammatory risk, RLR residual lipid risk, and RILR residual inflammatory and lipid risk. The baseline model included sex, age, history of diabetes, previous stroke or transient ischaemic attack, ACS-type, and GRACE risk estimates. *Reference: baseline model.

**Table S4**. Prediction error of biomarker-enhanced risk prediction models for 1-year MACE across residual risk groups.

|  | **Model** | **AIC (new model)** | **ΔAIC** | ***P* value*** |
| --- | --- | --- | --- | --- |
| **RLR** | **Individual biomarker models** |  |  |  |
|  | Bsl + hs-CRP | 669.83 | -2.28 | 0.039 |
|  | Bsl + JCAD | 672.72 | 0.61 | 0.24 |
|  | Bsl + LDL-c | 672.88 | 0.77 | 0.27 |
|  | **Combined models** |  |  |  |
|  | Bsl + JCAD + hs-CRP + LDL-c | 671.44 | -0.68 | 0.083 |
|  | Bsl + hs-CRP + LDL-c | 670.79 | -1.33 | 0.070 |
| **RIR** | **Individual biomarker models** |  |  |  |
|  | Bsl + JCAD | 615.06 | -1.53 | 0.060 |
|  | Bsl + hs-CRP | 616.32 | -0.27 | 0.13 |
|  | Bsl + LDL-c | 617.56 | 0.97 | 0.31 |
|  | **Combined models** |  |  |  |
|  | Bsl + JCAD + hs-CRP + LDL-c | 615.67 | -0.92 | 0.075 |
|  | Bsl + hs-CRP + LDL-c | 616.98 | 0.40 | 0.17 |
| **RILR** | **Individual biomarker models** |  |  |  |
|  | Bsl + JCAD | 404.90 | -1.59 | 0.058 |
|  | Bsl + hs-CRP | 407.52 | 1.03 | 0.32 |
|  | Bsl + LDL-c | 408.38 | 1.89 | 0.74 |
|  | **Combined models** |  |  |  |
|  | Bsl + JCAD + hs-CRP + LDL-c | 408.10 | 1.62 | 0.22 |
|  | Bsl + hs-CRP + LDL-c | 409.44 | 2.95 | 0.59 |

Bsl denotes baseline, HR hazard ratio, hs-CRP high-sensitivity C-reactive protein, GRACE Global Registry of Acute Coronary Events, JCAD Junctional Protein Associated with Coronary Artery Disease, LDL-c low-density lipoprotein cholesterol, MACE major adverse cardiovascular events, RIR residual inflammatory risk, RLR residual lipid risk, and RILR residual inflammatory and lipid risk. The baseline model included sex, age, history of diabetes, previous stroke or transient ischaemic attack, ACS-type, and GRACE risk estimates. *Reference: baseline model.

**Table S5**. Characteristics of RISK-PPCI study participants with available plasma samples.

|  | **Overall** |
| --- | --- |
|  | n = 139* |
| **Age ≥65 years** | 64 (47.1) |
| **Female** | 29 (21.3) |
| **JCAD (ng/mL)** | 1.64 [1.34, 2.05] |
| **TF (pg/mL)** | 26.84 [16.00, 101.65] |
| **TAFI (ng/mL)** | 66.34 [54.12, 88.70] |
| **PAI-1 (ng/mL)** | 3.49 [2.03, 6.73] |
| **BMI (kg/m^2^)** | 26.88 [23.89, 30.39] |
| **Smoking history** | 51 (37.5) |
| **eGFR (mL/min/1.73 m^2^)** | 84.99 [67.84, 94.39] |
| **Hx of DM** | 5 (3.7) |
| **Hx of hypercholesterolaemia** | 42 (30.9) |
| **Hx of hypertension** | 60 (44.1) |
| **FHx of CAD** | 60 (44.1) |
| **Hx of PAD** | 3 (2.2) |
| **Hx of stroke/TIA** | 6 (4.4) |
| **Hx of MI** | 11 (8.1) |
| **Hx of PCI** | 8 (5.9) |
| **Hx of CKD** | 5 (3.7) |
| **CRP (mg/L)** | 3.00 [1.00, 6.00] |
| **cTn (ng/L)** | 45.00 [19.25, 111.75] |
| **MACE at 1 year** | 8 (6.0) |

Continuous data are shown as median [25^th^-75^th^ percentiles] and categorical data as counts (%). *Out of 496 patients, 139 were available for biomarker analysis, with the corresponding study flow-chart being provided in Figure S1. BMI denotes body mass index, CAD coronary artery disease, CRP C-reactive protein, cTn cardiac troponin, DM diabetes mellitus, eGFR estimated glomerular filtration rate, FHx family history, Hx history, JCAD Junctional Protein Associated with Coronary Artery Disease, MACE major adverse cardiovascular events, MI myocardial infarction, PAI-1 plasminogen activator inhibitor-1, PAD peripheral artery disease, PCI percutaneous coronary intervention, TAFI thrombin activatable fibrinolysis inhibitor, TIA transient ischemic attack, and TF tissue factor.

**Table S6**. Linear associations of JCAD plasma levels with prothrombotic markers.

| **Outcome variable** | **Model** | **β (95% CI)** | ***P* value** |
| --- | --- | --- | --- |
| **TF** | Model 1: unadjusted | 1.203 (0.332, 2.075) | 0.007 |
|  | Model 2: + eGFR | 1.127 (0.224, 2.031) | 0.015 |
|  | Model 3: + eGFR + CRP | 1.106 (0.119, 2.093) | 0.028 |
| **TAFI** | Model 1: unadjusted | 0.724 (0.369, 1.078) | <0.001 |
|  | Model 2: + eGFR | 0.780 (0.408, 1.152) | <0.001 |
|  | Model 3: + eGFR + CRP | 0.722 (0.348, 1.096) | <0.001 |
| **PAI-1** | Model 1: unadjusted | 0.531 (-0.079, 1.141) | 0.088 |
|  | Model 2: + eGFR | 0.542 (-0.107, 1.191) | 0.101 |
|  | Model 3: + eGFR + CRP | 0.390 (-0.249, 1.028) | 0.229 |
| **LT** | Model 1: unadjusted | 0.386 (0.040, 0.733) | 0.029 |
|  | Model 2: + eGFR | 0.385 (0.036, 0.733) | 0.031 |
|  | Model 3: + eGFR + CRP | 0.398 (0.013, 0.783) | 0.043 |

Linear regression models were fitted to estimate the associations between JCAD and each outcome variable, i.e. TF, TAFI, PAI-1, and baseline lysis time. Based on directed acyclic graphs, three models were specified: Model 1 (unadjusted), Model 2 (adjusted for eGFR), and Model 3 (adjusted for eGFR and CRP). Results are expressed as regression coefficients (β) along with their corresponding 95% confidence intervals. CI denotes confidence interval, CRP C-reactive protein, eGFR estimated glomerular filtration rate (CKD-EPI formula 2009), JCAD Junctional Protein Associated with Coronary Artery Disease, LT baseline lysis time, PAI-1 plasminogen activator inhibitor-1, TAFI thrombin activatable fibrinolysis inhibitor, and TF tissue factor.
